# Massive transformation in FeNi nanopowders with nanotwin-assisted nitridation

**DOI:** 10.1038/s41598-022-07479-8

**Published:** 2022-03-07

**Authors:** Jian Wang, Yusuke Hirayama, Zheng Liu, Kazuyuki Suzuki, Wataru Yamaguchi, Kwangjae Park, Kenta Takagi, Hiroaki Kura, Eiji Watanabe, Kimihiro Ozaki

**Affiliations:** 1 Magnetic Powder Metallurgy Research Center, National Institute of Advanced Industrial Science and Technology, 2266-98 Anagahora, Shimoshidami, Moriyama, Nagoya 463-8560 Japan; 2 Innovative Functional Materials Research Institute, National Institute of Advanced Industrial Science and Technology, 2266-98 Anagahora, Shimoshidami, Moriyama, Nagoya 463-8560 Japan; 3 Advanced Research and Innovation Center, DENSO CORPORATION, 500-1, Minamiyama, Komenoki, Nisshin, Aichi 470–0111 Japan

**Keywords:** Magnetic properties and materials, Magnetic properties and materials

## Abstract

L1_0_-ordered FeNi alloy (tetrataenite), a promising candidate for rare-earth-free and low-cost permanent magnet applications, is attracting increasing attention from academic and industrial communities. Highly ordered single-phase L1_0_-FeNi is difficult to synthesis efficiently because of its low chemical order–disorder transition temperature (200–320 °C). A non-equilibrium synthetic route utilizing a nitrogen topotactic reaction has been considered a valid approach, although the phase transformation mechanism is currently unknown. Herein, we investigated the basis of this reaction, namely the formation mechanism of the tetragonal FeNiN precursor phase during the nitridation of FeNi nanopowders. Detailed microstructure analysis revealed that the FeNiN precursor phase could preferentially nucleate at the nanotwinned region during nitridation and subsequently grow following a massive transformation, with high-index irrational orientation relationships and ledgewise growth motion detected at the migrating phase interface. This is the first report of a massive phase transformation detected in an Fe–Ni–N system and provides new insights into the phase transformation during the nitriding process. This work is expected to promote the synthetic optimization of fully ordered FeNi alloys for various magnetic applications.

## Introduction

Permanent magnets are an integral part of the modern world and are indispensable in many critical technologies, including high-efficiency motors and generators, and medical equipment^1,2^. Current state-of-the-art permanent magnets mostly contain rare earth elements (Nd/Sm/Dy/Pr/La) owing to their high maximum energy product. However, such expensive, strategic, and insecure resources have raised severe concerns owing to their availability, extraction difficulties, and national security^3,4^. Thus, significant efforts have been made to develop alternative advanced rare-earth-free permanent magnets that deliver the same performance as rare-earth magnets, or at best exhibits an equal cost performance capability^5^.

Tetragonal L1_0_ FeNi (tetrataenite) is drawing considerable attention as a promising candidate for rare-earth-free permanent magnet materials^5–7^, owing to a high uniaxial magnetic anisotropy (0.6–1.3 MJ m^−3^), sufficiently large saturation magnetisation (1.6–1.65 T), and composition consisting of earth-abundant elements. Because of its low chemical order–disorder transition temperature (200–320 °C), the synthesis of single-phase, highly L1_0_-ordered FeNi alloys through conventional thermally activated processes is extremely challenging. Several attempts have been made to promote low-temperature atomic diffusion^8,9^ and/or directly fabricate L1_0_-ordered FeNi films via alternate monatomic layer deposition^10,11^. Recently, Goto et al*.* utilised a novel approach and artificially synthesized a L1_0_ FeNi with the highest reported degree of chemical ordering (*S* ~ 0.71) through nitrogen topotactic extraction from FeNiN powders^12^. However, bulk synthesis with a large volume fraction of the fully L1_0_-ordered FeNi phase has not been achieved in any laboratory to date. Thus, further fundamental studies on the metallurgical processing, phase formation, and microstructural changes to obtain ordered FeNiN precursor phase are essential, especially for the development of bulk L1_0_-ordered FeNi alloy-based permanent magnets.

**Figure 1:**
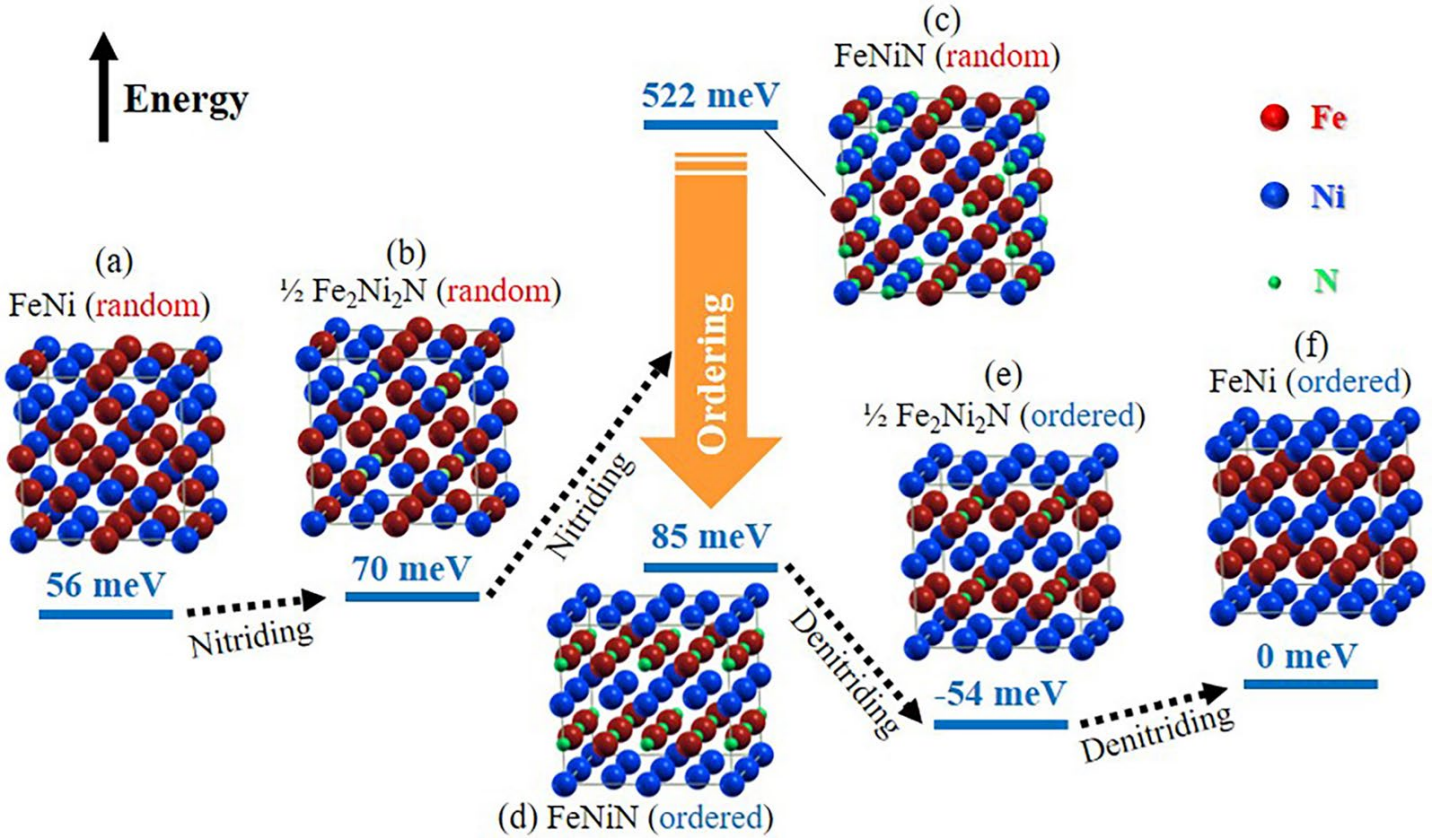
Energy diagram between the related states of the FeNi alloys during the nitriding/denitriding process. The random disordered FeNiN phase has a much higher energy than that of the L1_0_-ordered phase. Temporary and local over-nitriding of Fe_2_Ni_2_N may promote rearrangement of the metal elements. Red, blue, and green spheres denote iron, nickel, and nitrogen atoms, respectively.

Figure 1 illustrates a typical nitrogen topotactic reaction synthetic path^12^ from FeNi random alloy (A1-FeNi) (Fig. 1a) to the final L1_0_-ordered FeNi alloy (Fig. 1f), with the corresponding crystal model and calculated relative formation energy for each phase. The energy levels were calculated based on the ordered state of FeNi and the isolated N_2_ molecule (see “Methods”). The randomly disordered FeNi (A1-FeNi) alloy (Fig. 1a) is first nitrided into a partially randomly disordered Fe_2_Ni_2_N alloy (Fig. 1b), that is, until 1/4 of the octahedral sites are occupied by the nitrogen atoms and nickel atoms take the core sites. However, since the energy difference between the randomly disordered Fe_2_Ni_2_N phase and the ordered Fe_2_Ni_2_N (Fig. 1e) is only 124 meV, which appears too small to be the driving force behind the dynamic movement of metal atoms to fully order. Further nitriding Fe_2_Ni_2_N may lead to the formation of randomly disordered FeNiN alloy (Fig. 1c) with a much higher energy (522 meV vs. L1_0_-FeNi). Energy of FeNiN with L1_0_ type order (Fig. 1d) is, on the other hand, 437 meV lower than the random state, which seems to be a driving force for rearrangement of metal atoms. It is considered that a high-energy over-nitriding state, such as the randomly disordered FeNiN, can appear locally and temporarily on the particle surface or grain boundary of Fe_2_Ni_2_N under a certain condition. Then, local rearrangement of atoms occurs as a route to reduce the total energy of the system. The reaction process from random Fe_2_Ni_2_N to ordered FeNiN alloy is therefore expected to be extremely slow, which is consistent with the experimental results. Once an energetically stable FeNiN phase with a L1_0_ type order is established macroscopically, L1_0_-ordered FeNi (Fig. 1f) is readily obtained by the nitrogen topotactic extraction, considering that the metallic atom arrangement of ordered FeNiN is identical to that of L1_0_-ordered FeNi.

As demonstrated with Fig. 1, the most critical and time-consuming part of the nitrogen topotactic reaction route is related to the formation of the ordered FeNiN precursor phase. Therefore, in this work, we systematically studied the formation mechanism of the FeNiN precursor phase during the nitridation of FeNi nanopowders through detailed microstructure analysis. The results indicate a massive phase transformation from the random Fe_2_Ni_2_N to the ordered FeNiN phase, which is the first reported experimental demonstration of such an interface-controlled phase transformation in a Fe–Ni–N material system. This work provides valuable insights into the fundamental understanding of the phase transformation during nitridation, which may also promote further optimization for the development of L1_0_-ordered FeNi-based rare-earth-free permanent magnets.

## Results and discussion

### Microstructure evolution during nitridation

**Figure 2:**
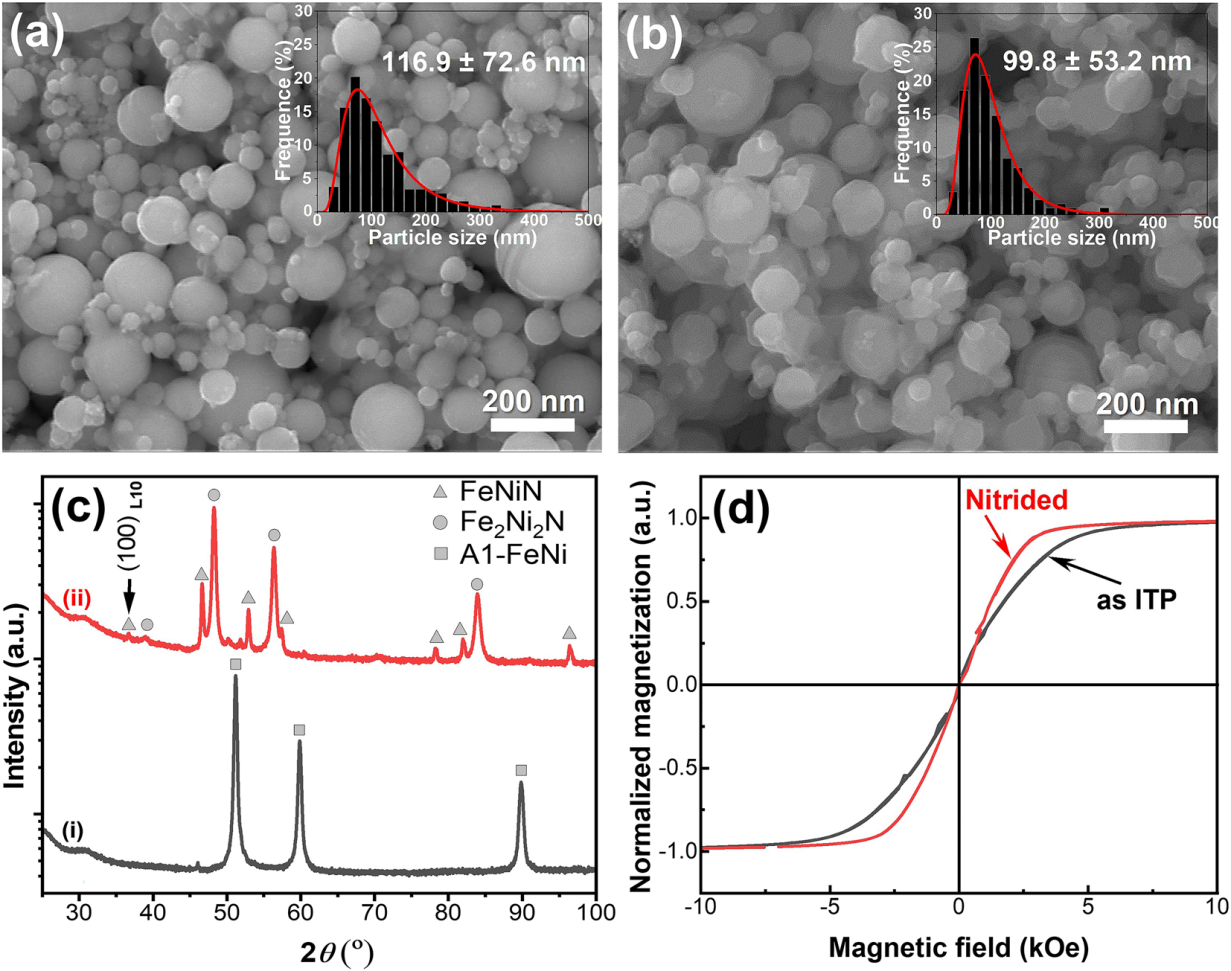
Microstructure evolution before and after the nitriding process. (**a**,**b**) FE-SEM images and (**c**) XRD profiles of the (**a**,**c**-**i**) as induction thermal plasma processed (as ITP) and (**b**,**c-ii**) nitrided FeNi NPs. (**d**) Magnetic hysteresis curves (room temperature) of the as ITP and nitrided FeNi NPs. Insets in the FE-ESEM images show the corresponding particle size distribution.

The FeNi nanopowders (NPs) were synthesized using a novel low oxygen induction thermal plasma (LO-ITP) system. The as ITP synthesized NPs were then reduced under a hydrogen gas flow to remove the possible oxide surface. Finally, the FeNi NPs were nitrided under an ammonia gas flow (see “Methods”). To investigate the formation mechanism of the FeNiN precursor phase in the FeNi NPs during nitridation, we conducted a detailed microstructure analysis of NPs in an intermediate state, that is, a mixture of the Fe_2_Ni_2_N parent and FeNiN product phases. Figure 2a,b shows the morphology with scanning electron microscopy (SEM) images of the as ITP processed and nitrided FeNi NPs, respectively. Over 500 NPs were counted for the particle size distribution (insets in Fig. 2a,b), and the mean particle size is approximately 116.9 ± 72.6 and 99.8 ± 53.2 nm for the as ITP processed and nitrided FeNi NPs, respectively. Figure 2c presents the X-ray diffraction (XRD) patterns of (1) as ITP processed and (2) nitrided FeNi NPs. As indicated, the as ITP processed FeNi NPs possess a single A1-FeNi phase, whereas the nitrided FeNi NPs show a mixture of Fe_2_Ni_2_N and FeNiN phases. As indexed in Fig. 2c-ii, the (100)_L10_ superlattice peak around 37° was detected (see Supplementary Fig. S1). Meanwhile, the presence of both (002)_FeNiN_ peak (around 57°) and (200)_FeNiN_ peak (around 79°) indicate a tetragonal lattice distortion of the FeNiN phase. These features reveal that the FeNiN phase detected in current work belongs to the L1_0_-ordered structure while the Fe_2_Ni_2_N phase belong the cubic fcc structure. Rietveld analysis using the RIETAN-FP software^13^ revealed approximately 80% of the Fe_2_Ni_2_N phase and 20% of the FeNiN phase in the nitrided FeNi NPs. Such an intermediate state with mixed phases during nitridation can serve as a suitable material platform for investigating the formation mechanism of the FeNiN precursor phase. Figure 2d demonstrate the room temperature nominalized magnetization curves of the as ITP and nitrided FeNi NPs. The powder samples were sealed in the Ar filled plastic tube to avoid oxidation and the paramagnetic contribution was removed from the raw data (see “Methods”). As can be seen, both the as ITP and nitrided FeNi NPs are magnetic soft with small coercivity which indicate the small magnetic anisotropy of the A1-FeNi, Fe_2_Ni_2_N and FeNiN phases.

### Nucleation of the FeNiN phase in the nanotwinned region

**Figure 3:**
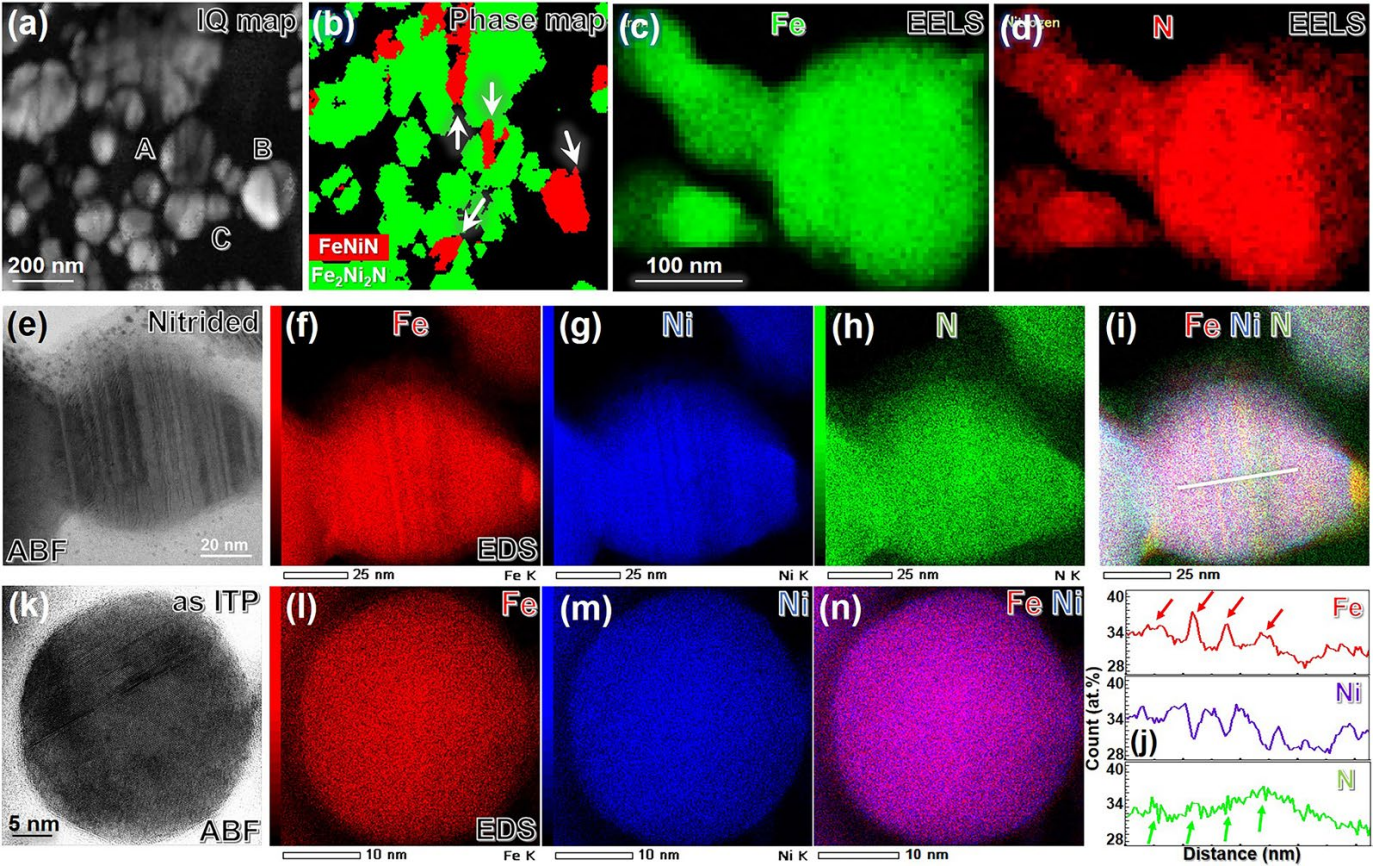
Correlation between FeNiN phase and nanotwins. (**a**) TKD image quality (IQ) map and (**b**) TKD phase map of the nitrided FeNi NPs. The interfaces between the FeNiN (in red) product phases and Fe_2_Ni_2_N (in green) parent phases are indicated with the white arrow in the phase map (**b**). EELS elemental maps of (**c**) Fe and (**d**) N of nitrided FeNi NPs marked as “**B**” in the IQ map (**a**). Angular bright-field STEM images (**e**, **k**) and EDS elemental maps (**f**–**i** and **l**–**n**) of the (**e**–**i**) nitrided and (**k**–**n**) as ITP processed FeNi NPs. (**j**) A line elements scan profile of the nitrided FeNi NP across the nanotwinned region as the line marked in the composite map (**i**).

To investigate the formation mechanism of the FeNiN precursor phase, it is important to firstly identify where and how the FeNiN phase distributed in the nitrided FeNi NPs. Since Fe_2_Ni_2_N and FeNiN phase belongs to space groups with distinct lattice symmetry (Pm-3 m and P4/mmm for the Fe_2_Ni_2_N and FeNiN phase, respectively) and have different lattice constants (*a* = 0.377 nm for the Fe_2_Ni_2_N phase vs. *a* = 0.400 nm, *c* = 0.371 nm for the FeNiN phase). Thus, it is possible to distinguish between the two phases using transmission Kikuchi diffraction (TKD) analysis. Figure 3a,b show the TKD image quality and phase maps, respectively. The clear colour contrast in the TKD phase map (Fig. 3b) confirms a mixture of the FeNiN (red) and Fe_2_Ni_2_N (green) phases in the nitrided FeNi NPs, which is consistent with the XRD result (Fig. 2c-ii). Interestingly, as indicated by the white arrows in Fig. 3b, the FeNiN and Fe_2_Ni_2_N phases in the nitrided FeNi NPs usually share sharp/straight boundaries.

Another approach considered to distinguish the FeNiN and Fe_2_Ni_2_N phases is the different nitrogen concentration in the Fe_2_Ni_2_N (20.0%) and FeNiN (33.3%) phases. Specifically, the visible contrast from the electron energy loss spectroscopy (EELS) and energy dispersive X-ray spectroscopy (EDS) mapping of nitrogen can help accurately identify where and how the FeNiN phase distributed in the nitrided FeNi NPs. Figure 3c,d show the representative EELS maps of the nitrided FeNi NPs, marked as “B” in Fig. 3a. With a high sensitivity to the light elements, the EELS map of the nitrogen distribution in Fig. 3d shows that the left part of “B” has a relatively higher nitrogen concentration than the remaining right part, sharing a boundary which is consistent with the TKD phase map in Fig. 3b. Interestingly, the nitrogen distribution feature detected here is different from the conventional core/shell structure reported in the nitrided Ti and Fe NPs^14,15^, implying a distinct nitrogen diffusion process and special attention to the twin boundary in the current FeNi NPs.

Figure 3e shows the annular bright-field scanning transmission electron microscopy (ABF-STEM) image of the FeNi NP marked as “C” in Fig. 3a. The multiple bright/dark stripe diffraction contrasts in the ABF-STEM image, which can be due to the chemical composition fluctuation and/or the lattice direction change across the twin boundaries (TBs). Later high-resolution TEM observation confirmed such feature as a high-density nanotwin substructure in the nitrided FeNi NPs. The low-magnification TKD grain boundary maps (refined rotation angle: 65°–75°, shown in Supplementary Fig. S2) reveal that nearly 49.5% of the as ITP processed and ~ 38.1% of the nitrided FeNi NPs contained nanotwin substructure. Such intensive nanotwins in the FeNi NPs may have formed during the quenching of the ITP process (quenching twins or mechanical twins^16^), induced by the thermal strain energy field relief or nitridation process (annealing twins^17^), a consequence of the recrystallisation/phase transformation of face-centred cubic (fcc) metals with low stacking fault energies. Surprisingly, we observe significant solutes segregation at the TBs detected in the corresponding EDS maps (Fig. 3f–i). The line scan profiles across the nanotwinned regions (Fig. 3j) indicate a clear enrichment of Fe, and N while depletion of Ni occurs along the nanotwin regions (indicated by the arrows in Fig. 3j). With further detailed high-resolution TEM observation and EDS mapping (see Supplementary Fig. S3), it is confirmed that solutes segregant is mainly located at the TBs. More solutes segregation examples (EDS maps) can be found in Supplementary Fig. S4. To confirm whether such solutes segregation initially occurs during the thermal plasma process or the nitridation process, EDS mapping was also checked for the as ITP processed FeNi NPs. Figure 3k–n shows the representative ABF-STEM images and corresponding EDS maps of the as ITP processed FeNi NPs. Although the typical nanotwins were observed in Fig. 3k, there was no visible solute segregation in the corresponding EDS maps (Fig. 3l–n) (see additional example in Supplementary Fig. S5), which indicates that solute segregation mostly raised during the nitridation process. Grain boundary segregation and solute diffusional phenomena are well reported interface characteristics^18,19^. In contrast, coherent TBs are dislocation-free and have perfectly aligned atomic stacks, resulting in low interfacial energies compared to grain boundaries; thus, strong solute segregation is not initially expected at coherent TBs. However, several recent reports on different alloy systems have shown solute segregation along TBs and proved it is associated to the migrating heterophase interface boundary, driven by minimizing the intrinsic stacking fault, elastic strain, and/or total energy in the system^20–22^. On the other hand, it is also worth notice that solutes accumulation at the TBs can also act as Cottrell clouds surrounding the twins and thereby restrict further slip of the dislocations which was well discussed as the strength enhancement mechanism for the twinned soft materials^23–25^. Since the strong twin boundary-dislocation pinning interaction, the twin growth and/or detwinning process which determined by the dislocation motion will become time dependent. It could be one of possible reasons why the synthesis of the FeNiN precursor phase is the most time-consuming part of the nitrogen topotactic reaction route.

**Figure 4:**
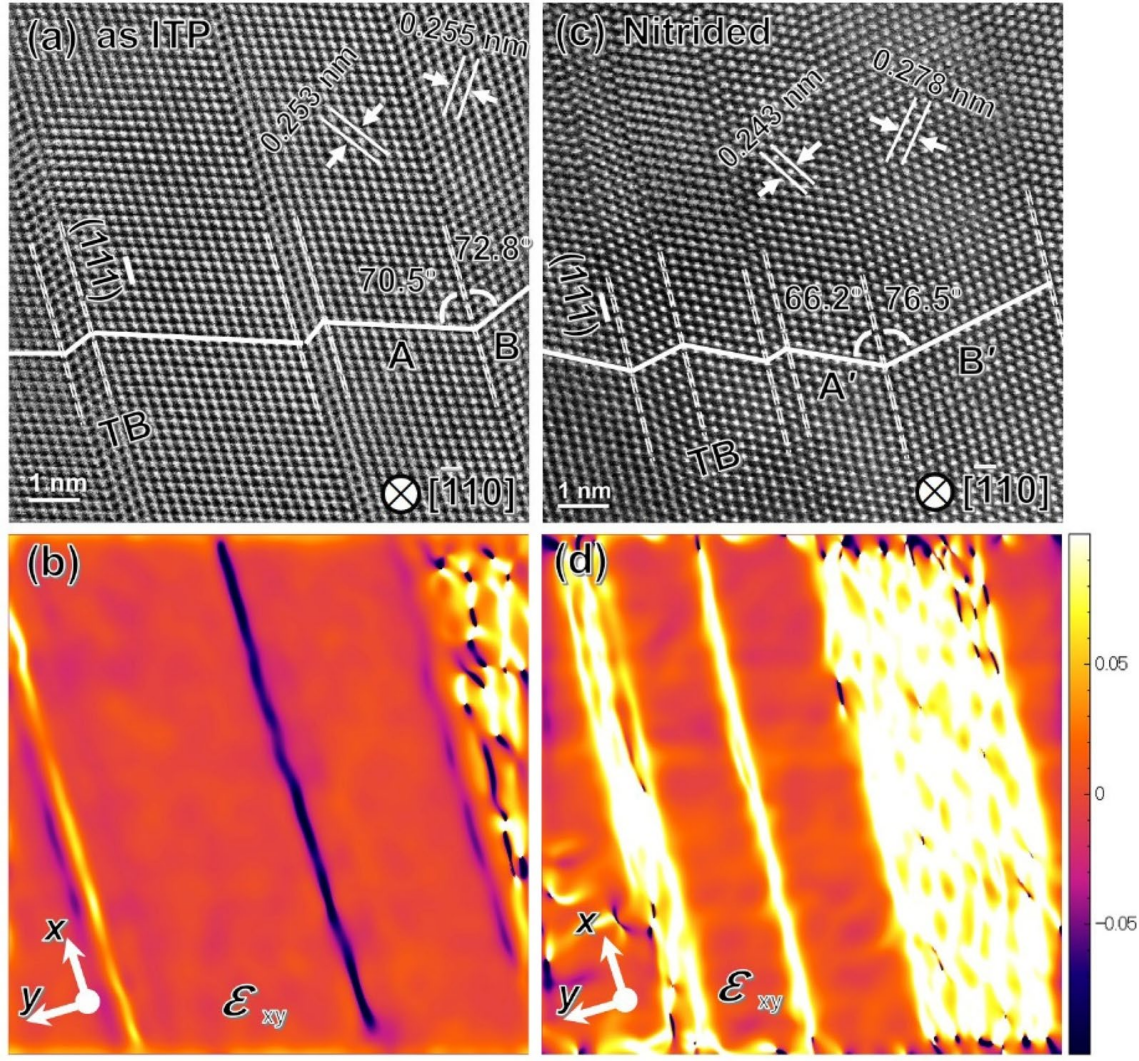
Shear stress induced lattice distortion and incoherence at the nanotwinned region. (**a**,**c**) High-resolution HAADF-STEM images demonstrating parallel multi-nanotwin TBs along the ($11\overline{1}$) plane are indicated by the dashed lines and (**b**,**d**) corresponding GPA strain maps showing the shear strain (strain tensor component *ε*_xy_) of the (**a**,**b**) as induction thermal plasma processed and (**c**,**d**) nitrided FeNi NPs. The colour scale bar for strain ranges from − 10% (dark blue) to + 10% (white).

Figure 4a,c presents the atomic-resolution angle annular dark-field (ADF)-STEM images viewed along the [$\overline{1}10$] zone axis 1to elucidate the detailed features of the atomic stacking and TBs of the as ITP processed and nitrided FeNi NPs, respectively. From the ADF-STEM images, the stacking faults parallel to the ($11\overline{1}$) plane creates nanotwin substructures in the matrix with a width varying from 2 (~ 0.5 nm) to 16 atomic layers (~ 3.5 nm). The nanotwin substructures are also confirmed by the typical two-set patterns presented in the fast Fourier transform (FFT) images in Supplementary Fig. S6. For the as ITP processed A1-FeNi NP in Fig. 4a, the (110) interplanar spacing in the nanotwinned region (marked as “B” in Fig. 4a) is ${d}_{110}^{\mathrm{twin}}$= 0.255 nm while it is ${d}_{110}^{\mathrm{matrix}}$= 0.253 nm for the matrix region (marked as “A” in Fig. 4a). Considering the spatial resolution of the TEM facility and inherent error of manually operation, such small difference in lattice spacing is within the standard deviation error. On the other hand, remarkable (110) interplanar spacing expansion is detected in the nitrided FeNi NP with ${d}_{110}^{\mathrm{twin}}$= 0.278 nm in the nanotwinned region (marked as “B′ ” in Fig. 4c), while ${d}_{110}^{\mathrm{matrix}}$= 0.243 nm in the matrix region (marked as “A′ ” in Fig. 4c). Furthermore, the (111) planes in both the matrix and nanotwinned regions of the as ITP FeNi NPs follow an approximate mirror symmetry manner with respect to the ($11\overline{1}$) twining plane1 (as indicated in Fig. 4a, 70.5° vs. 72.8°). However, such mirror symmetry serious degraded in the nitrided FeNi NPs, as indicated in Fig. 4c (66.2° vs. 76.5°) which finally result in an “incoherent” twin boundaries with much higher boundary energy than a coherent twin boundaries^26^. To estimate the lattice distortion/strain distribution at the nanotwinned regions, a geometric phase analysis (GPA) was conducted to reconstruct strain maps from the corresponding atomic-resolution ADF-STEM images (see “Methods”). Figure 4b,d shows the shear strain (*ε*_xy_) field maps of the as ITP processed and nitrided FeNi NPs, respectively. Although the atomic strain analysis is affected by camera resolution, the difference in the shear strain maps is substantial, indicating the qualitative difference in the lattice distortion between the twin and matrix regions especially for the nitrided FeNi NPs. As the weak contrast in the strain map (Fig. 4b) can be attributed to the small strain in the as ITP FeNi NPs. The bright contrast in the shear strain maps which indicating positive shear strain suggests that the crystal units are significantly stretched along the [110] direction (which is close to xy direction) and compressed in the [002] direction for the nitrided FeNi NPs (Fig. 4d). Furthermore, a comparison of the interplanar spacing of the (110) and (002) planes between the matrix and nanotwinned regions of the nitrided FeNi NPs (spacing calculated from Fig. 4c) reveals a + 12.8% expansion along the [110] direction and –10.2% shrinkage along the [002] direction in the nanotwinned region (the bright region in Fig. 4d). The severe shear strain triggers the dislocation accumulations in the nanotwinned region, which eventually lead to significant lattice distortions as well as the detected ($11\overline{1}$) plane 1expansion. Similar phenomena has been reported by Wu et al*.* in the FeCoCrNi high-entropy alloy^27^. In addition, the chemical composition (Fe:Ni:N, at.%) of the expended/nanotwinned region estimated from EDS elements maps is about 36.2: 30.2: 33.6 which is 33.8: 36.7: 29.5 for the matrix region. As the strain induced lattice distortion is considered dominant in the nitrided FeNi NPs, the different lattice spacing may also partially originated from the chemical composition fluctuation across the nanotwin region (as shown in Fig. 3j).

Thus, we have experimentally demonstrated that: (a) twins are typical features present in both the as ITP processed (~ 49%) and nitrided FeNi NPs (~ 38%); (b) in some of the nanotwinned regions of nitrided FeNi NPs, there is a visible enrichment of Fe and N solutes while a depletion of Ni at the TBs. The enriched Fe at the TBs will further attract more nitrogen due to its relatively smaller formation energy of iron-nitride than nickel-nitride^28^; (c) the nanotwinned region of nitrided FeNi NPs suffers from severe shear strain (*ε*_xy_ ~ 10%) which consequently induces a significant lattice distortion. Thus, it is believed that such nanotwinned regions with relatively higher nitrogen concentration and low-symmetry crystal will be energy favourable to promote the nucleation of the FeNiN product phase.

### Massive transformation of Fe_2_Ni_2_N to FeNiN phase

As mentioned previously, the FeNiN product phase possesses different nitrogen concentrations and crystal structures compared to the Fe_2_Ni_2_N parent phase; thus, one can detect sharp and sometimes straight phase boundaries in nitrided FeNi NPs (as illustrated in Fig. 3b,d) in which the phase transformation is not fully complete. Therefore, after the preferential nucleation of the FeNiN product phase in the nanotwinned region, it is important to understand how this new product phase grow into the Fe_2_Ni_2_N parent matrix. To clarify this critical question, a detailed microstructure analysis focusing on the FeNiN/Fe_2_Ni_2_N phase boundaries was performed.

**Figure 5:**
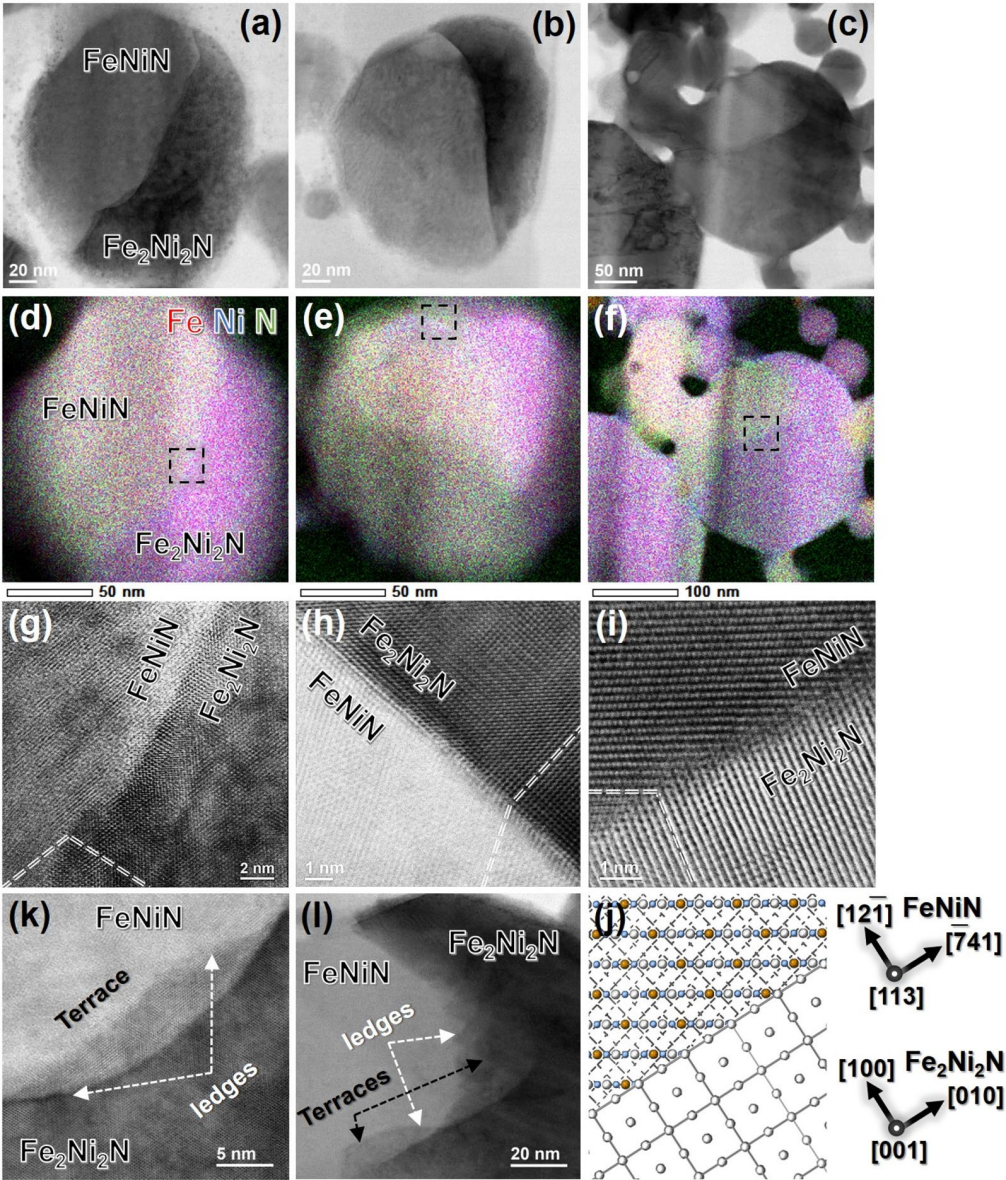
Coherent/incoherent Fe_2_Ni_2_N/FeNiN phase interfaces with ledgewise growth features. (**a–c**) ABF-STEM images and (**d–f**) the corresponding combined (Fe, Ni, and N) elements distribution mapping of the nitrided FeNi NPs. (**g–i**) High-resolution ABF-STEM images focusing on the detailed microstructure of the FeNiN/Fe_2_Ni_2_N interfaces. The observed areas are indicated by the dashed squares marked in the corresponding element distribution maps (**d**–**f**). The high-index/irrational OR developed at the FeNiN/Fe_2_Ni_2_N coherent (**h**)/incoherent (**g**,**i**) interfaces were indicated by the dashed line and one representative high-index orientation relationship present in (**i**) is schematised in (**j**). (**k**,**l**) ABF-STEM images of nitrided FeNi NPs with terraces/ledges demonstrating the ledgewise growth motion of the FeNiN/Fe_2_Ni_2_N phase interface in (**a**) and (**c**), respectively.

Figure 5a–c shows the ABF-STEM images of the nitrided FeNi NPs in which the distinct bright/dark phase contrast indicates clear FeNiN/Fe_2_Ni_2_N phase boundaries. Figure 5d–f shows the corresponding EDS composite (Fe (red)-Ni (blue)-N (green)) maps. The light yellow/pink contrast illustrates a relatively N-rich/-poor zone, which was later identified corresponding to the FeNiN product/Fe_2_Ni_2_N matrix phase by matching the lattice spacing and indexing in the FFT patterns and the atomic-resolution ADF-STEM image (see Supplementary Fig. S7). The clear, sharp, and sometimes straight phase boundaries are consistent with the phase/elements distribution characteristics observed in Fig. 3b,d. With detailed microstructure characterisation (Fig. 5g–i) of the FeNiN/Fe_2_Ni_2_N phase boundaries, it is surprising that the FeNiN product phase usually develops a high-index/irrational orientation relationship (OR) with the Fe_2_Ni_2_N parent matrix. The FeNiN/Fe_2_Ni_2_N phase boundary displayed in Fig. 5g (corresponding to the dash squared region in Fig. 5d) seems to show a high-index/irrational OR at the interface with the Fe_2_Ni_2_N phase on a [110] zone axis, while the FeNiN phase is tilted slightly away from the exact zone axes. In Fig. 5h (corresponding to the dash squared region in Fig. 5e), the FeNiN/Fe_2_Ni_2_N phase boundary demonstrates a sharp/fully epitaxial coherent interface with the Fe_2_Ni_2_N phase on a [100] zone axis, while the FeNiN phase is tilted slightly away from the exact zone axes. Finally, in Fig. 5i (corresponding to the dash squared region in Fig. 5f), the FeNiN/Fe_2_Ni_2_N phase boundary demonstrates an atomic rough incoherent interface with the FeNiN phase on a [113] zone axis, while the Fe_2_Ni_2_N phase is tilted slightly away from the [001] zone axes. The possible high-index OR developed at the FeNiN/Fe_2_Ni_2_N interface is illustrated in Fig. 5j: ($12\overline{1}$)_FeNiN_ [$\overline{7}41$]_FeNiN_ || (100)_Fe2Ni2N_ [010]_Fe2Ni2N_. Such a high-index OR results in a small lattice misfit (1.8%) at the interface which favours a lower strain/interfacial energy. The results indicate that it is possible for two phases with a high-index OR, and to obtain some degree of atomic matching across the high-index interface planes by selecting a suitable interface plane in the nitrided FeNi NPs. However, some occasional planes apparently have no matching planes on the opposing side of the boundary, makes such an interface partly coherent in the plane-to-plane geometry. The absence of a lattice correspondence defines such a FeNiN/Fe_2_Ni_2_N interface in Fig. 5i as structurally incoherent. Thus, the FeNiN/Fe_2_Ni_2_N phase boundaries in the nitrided FeNi NPs present both a structurally commensurate (coherent) and incommensurate (incoherent) interface, with a variable degree of commensurability depending on the orientation and planarity of a particular interface.

Figure 5k,l shows the relatively low-magnification ABF-STEM images of the FeNiN/Fe_2_Ni_2_N interfaces displayed in Fig. 5a,c, respectively. It demonstrates the typical terraces/ledges featured at the interfaces, implying that the growth of the FeNiN product phase, accompanied by the prorogation of the interfaces, follows a ledge mechanism^29,30^. This kind of ledgewise growth motion of the interface has been reported in the FeNi^31,32^, MnAl^21,33^, and TiAl^34,35^ alloy systems associated with the massive transformation process. It has been experimentally demonstrated that massive transformations involve a special crystallographic OR between the parent and the product phases, leading to coherent nucleation. This is followed by growth, in which the interface changes from coherent to partly coherent, or even to a high-index irrational interface, and migrates by a ledge mechanism^29,30^, which is principally accomplished by short-range atom jumps that occur almost continuously across the structurally incoherent interfaces^21,34^. The massive γ to α transformation was previously reported in the FeNi binary alloy system (with a Ni concentration ≤ 15%)^36–38^. The present results of elements segregated at the TBs (Fig. 3i,j), the high-index OR developed at the FeNiN/Fe_2_Ni_2_N interface (Fig. 5g–j), and the ledgewise growth motion of the FeNiN/Fe_2_Ni_2_N interface (Fig. 5k,l) agrees well with the typical characteristics of a massive transformation, indicating that the FeNiN phase is most likely formed through a massive transformation from the Fe_2_Ni_2_N matrix phase during the nitridation process. And this is the first experimental demonstration of a massive transformation in the Fe–Ni–N system. Based on our experimental results, one possible formation route for the FeNiN precursor phase during the nitridation process is proposed as follows: first, the FeNiN product phase initially energetically favours to nucleation at the nanotwinned region in the Fe_2_Ni_2_N parent matrix. Then the FeNiN/Fe_2_Ni_2_N interface develops a high-index OR through the short-range atoms jump across the interface to minimise the strain/interface energy. Finally, the massive Fe_2_Ni_2_N to FeNiN phase transformation is accomplished through the migration of the high-index OR interfaces following a ledge mechanism. In some nanotwinned regions, solutes segregation at the TBs during the massive transformation may occur and the block the dislocation motion which will restrict the FeNiN/Fe_2_Ni_2_N interface migration and finally leads to a time-consuming phase transformation process.

## Conclusion

In this work, we investigated the formation mechanism of the FeNiN phase (the precursor phase of the L1_0_-ordered FeNi for synthesise of rare-earth-free permanent magnets) in FeNi NPs during nitridation process. We found that the intensive nanotwins in the nitrided FeNi NPs possess a distorted lattice and relatively higher nitrogen concentration, which could provide preferential nucleation sites for the FeNiN product phase in the Fe_2_Ni_2_N parent matrix. Furthermore, detailed microstructure analysis revealed that the growth of the FeNiN product phase followed a massive transformation with high-index irrational orientation relationships and ledgewise growth motion characteristics detected at the FeNiN/Fe_2_Ni_2_N migrating interface. Based on the results, we delineated a potential formation route of the FeNiN precursor phase in the FeNi NPs during nitridation, which could contribute to the basic understanding of this mechanism and promote further optimization of the synthesis of bulk ordered FeNi alloys for various magnetic applications.

## Methods

### Materials synthesis

FeNi nanoparticles (NPs) were synthesized using a low oxygen induction thermal plasma (LO-ITP) system (TP-40020NPS, JEOL Co., Ltd.) and a powder feeding system (TP-99010FDR, JEOL). The detailed configuration information of the LO-ITP system can be found in Ref.^39^. Fe (*D* = 3–5 μm, 99.9% purity) and Ni (*D* = 3–5 μm, 99.9% purity, Kojundo Chemical Lab. Co., Ltd., Japan) powders were used as starting materials and mixed with a mole ratio of Fe:Ni = 1:1. The ITP was generated using a radio frequency generator with a power of 6 kW and a frequency of 13.56 MHz. G1 grade Ar gas (oxygen level of less than 0.1 ppm) was used as the plasma gas with a flow rate of 35 L/min and as a powder feeding gas with a flow rate of 3 L/min using a powder feeding system (TP-99010FDR, JEOL Ltd.). The mixed powders were continuously introduced from the top of the thermal plasma using a powder feeder for 10 min. After the ITP process, the processed NPs were collected from the water-cooled chamber wall and handled in a glovebox with an oxygen level below 0.5 ppm.

The as ITP processed FeNi NPs were first reduced in an electric furnace at 400 °C under a hydrogen gas flow at 1 L/min for 2 h. Then, the processed NPs were nitrided at 350 °C under a large amount of ammonia gas flowing at a rate of 2 L/min for 16 h. To prevent oxidation, most of the experiments and evaluations in this work were carried out under a low oxygen atmosphere (glovebox) without exposure to the atmosphere, except when briefly removing the samples for characterisation by SEM and transmission electron microscopy (TEM).

### Theoretical calculation

The energies of the randomly disordered and L1_0_-ordered FeNi alloys and their nitrides were calculated using the spin-polarised density functional theory formalism^40,41^. Ultrasoft-type pseudopotentials with a plane wave basis set were used to describe the electronic structure within the generalized-gradient approximation (GGA) using the parameters by Perdew, Burke, and Ernzerhof (PBE)^42^. The computations were carried out using the PWscf code implemented in the QUANTUM ESPRESSO package^43,44^. The *k*-points for the Brillouin zone integration were sampled on a 4 × 4 × 4 grid according to the Monkhorst–Pack scheme^45^. The kinetic energy cut-offs for the wavefunctions and charge density were 90 Ry and 1080 Ry, respectively. The self-consistent field convergence was determined by the threshold value of 10^–6^ Ry for the estimated energy error.

A 2 × 2 × 2 supercell (32 metal atoms) of the FeNi fcc or tetragonal (fct) unit cells (four metal atoms) was used to express the random alloy state. Repeat random assignments of each half of the 32 transition element sites to Fe and Ni was achieved using computer-generated pseudorandom numbers. One FeNi arrangement was unbiasedly selected from those with an order parameter of S = 0 and was used in the calculation of the random states. For structural relaxation, we did not perform a full optimisation for each individual atom, but only optimised the lattice constants. The relative positions of the metal atoms within the unit cell were fixed at the ideal position of the fcc or fct lattice. The energies of each state were defined as the formation energies from the lowest energy polytypes of the constituent elements, namely bcc-Fe, fcc-Ni, and an isolated N_2_ molecule.

### Structural characterization

The phases were identified using an X-ray diffractometer (PANalytical, Empyrean, Co-*K*α, λ = 1.78901 Å). The particle morphology and size distribution were estimated from images captured using a field-emission scanning electron microscope (FE-SEM, JSM-7800F, JEOL).

Nanoscale crystallographic/phase characterisation was performed using TKD analysis. TKD (also known as transmission electron backscatter diffraction (t-EBSD)) is an electron diffraction method used in SEM that offers a dramatic improvement in spatial resolution (2–5 nm^46^) over traditional EBSD, which is comparable to nano-diffraction techniques in TEM^47^. The epoxy-embedded FeNi NPs were first sliced (cross-section sample) and fixed on a Cu grid via an FEI Scios focused ion beam–scanning electron microscopy (FIB-SEM) facility. A conventional EBSD detector (EDAX DigiView 5 with EDAX TEAM software) was used for the TKD analysis. TSL-OIM 7 software was used to post-process the TKD raw data.

The high-resolution scanning transmission electron microscopy (STEM), EELS, and EDS mapping were carried out on identical Cu grid specimens by employing an atomic-resolution analytical electron microscope JEM‐ARM200F (JEOL Ltd. Tokyo, Japan). The system includes an imaging Cs corrector and is operated at an acceleration voltage of 200 kV. The lattice spacing was estimated by using Gatan’s *DigitalMicrograph* ™ software with the drift corrected HRTEM image. The inverted FFT image was firstly obtained after selecting specific diffraction spots in the FFT image (deduced from HRTEM image). Then, a straight line was draw in a perpendicular position to the represented atomic layers in the inverted FFT image and got the corresponding line profile. The final lattice spacing is calculated by averaging the peak distance (more than 100 peaks involved) which represent the d-spacing in the line profile.

The magnetic properties were measured using a physical property measurement system equipped with a vibrating sample magnetometer (PPMS-VSM/Quantum Design, Dynacool). The maximum field for the measurement was 90 kOe. Since the VSM measurements were performed outside glove box, the powder samples were sealed in Ar gas filled plastic tube to avoid the oxidation.

### Strain mapping

The strain distribution around the nanotwinned regions of FeNi NPs was analysed using a geometric phase analysis (GPA)^48^ on the individual atomic-resolution HAADF-STEM images. GPA is normally used to measure in-plane displacements and/or strains at the nanometre scale within an atomic-resolution TEM field of view using a FFT-based analysis of the local lattice parameters^48,49^. Before GPA, over 20 aberration-corrected STEM images were acquired from the same region and the corresponding atomic positions were averaged/shift corrected to minimise the possible influence of vibration during scanning on the strain maps. Then, with the two-dimensional atomic arrangement shown in the STEM images, the tensorial distortion is defined as^48^

1 $$e=\left(\begin{array}{cc}{e}_{xx}& {e}_{xy}\\ {e}_{yx}& {e}_{yy}\end{array}\right)=\left(\begin{array}{cc}\partial {u}_{x}\left/ \partial x\right.& \partial {u}_{x}\left/ \partial y\right.\\ \partial {u}_{y}\left/ \partial x\right.& \partial {u}_{y}\left/ \partial y\right.\end{array}\right)$$

where **u** = (*u*_x_, *u*_y_) denotes the displacement field. In addition, the shear strain in this study is defined as:

2 $${\varepsilon }_{xy}={\varepsilon }_{yx}=1/2\left(\partial {u}_{x}\left/ \partial y\right.+\partial {u}_{y} \left/ \partial x\right.\right)$$

The GPA in this work was conducted with an open-source software Strain++^49^.

## Supplementary Information

Supplementary Figures.
