# Supplementary material for: Massive transformation in FeNi nanopowders with nanotwin-assisted nitridation
Source: Sci Rep. 2022 Mar 7;12:3679. doi: 10.1038/s41598-022-07479-8 (PMC8901742; doi:10.1038/s41598-022-07479-8)
Supplement: Supplementary file 1 — Supplementary Figures. [file 41598_2022_7479_MOESM1_ESM.docx]

**Supplementary Information**

Massive transformation in FeNi nanopowders with nanotwin-assisted nitridation

Jian Wang^1*^, Yusuke. Hirayama^1*^, Zheng Liu^2^, Kazuyuki Suzuki^1^, Wataru Yamaguchi^1^, Kwangjae Park^1^, Kenta Takagi^1^, Hiroaki Kura^3^, Eiji Watanabe^3^ and Kimihiro Ozaki^1^

*^1^ Magnetic Powder Metallurgy Research Center, National Institute of Advanced Industrial Science and Technology, 2266-98 Anagahora, Shimoshidami, Moriyama, Nagoya, 463-8560, Japan.*

*^2^ Innovative Functional Materials Research Institute, National Institute of Advanced Industrial Science and Technology, 2266-98 Anagahora, Shimoshidami, Moriyama, Nagoya, 463-8560, Japan.*

*^3^ Advanced Research and Innovation Center, DENSO CORPORATION, 500-1, Minamiyama, Komenoki, Nisshin, Aichi, 470–0111, Japan*

^*^e-mail: wang.jian86@aist.go.jp; hirayama.yusuke@aist.go.jp


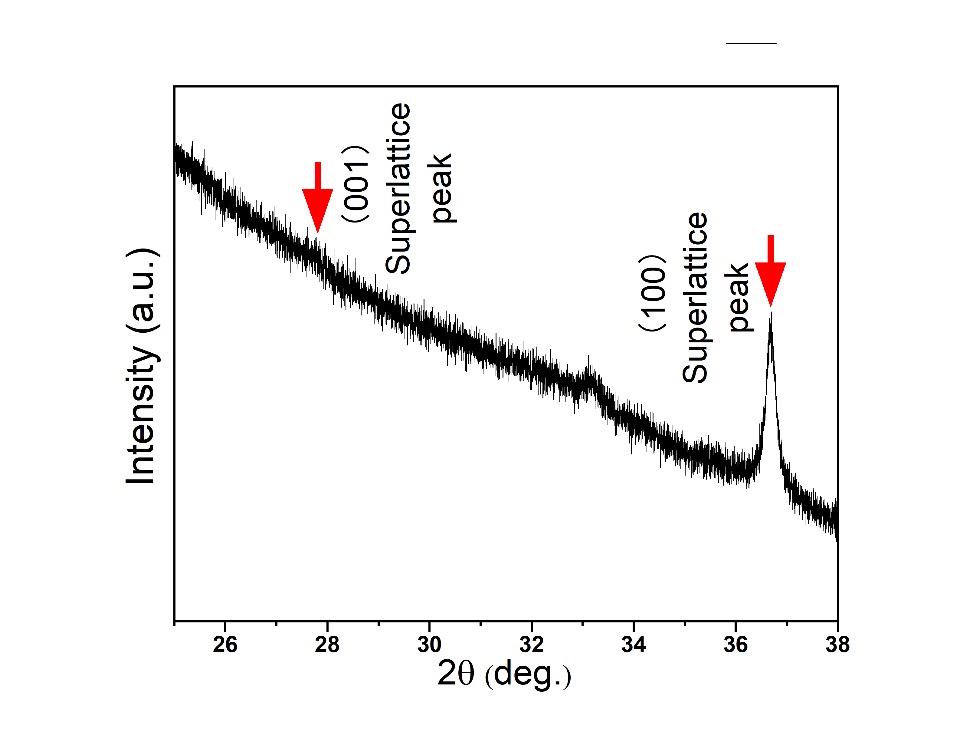


**Supplementary Figure S1 |** Low angle region XRD profiles of the nitrided FeNi NPs with single-crystal silicon sample holders.


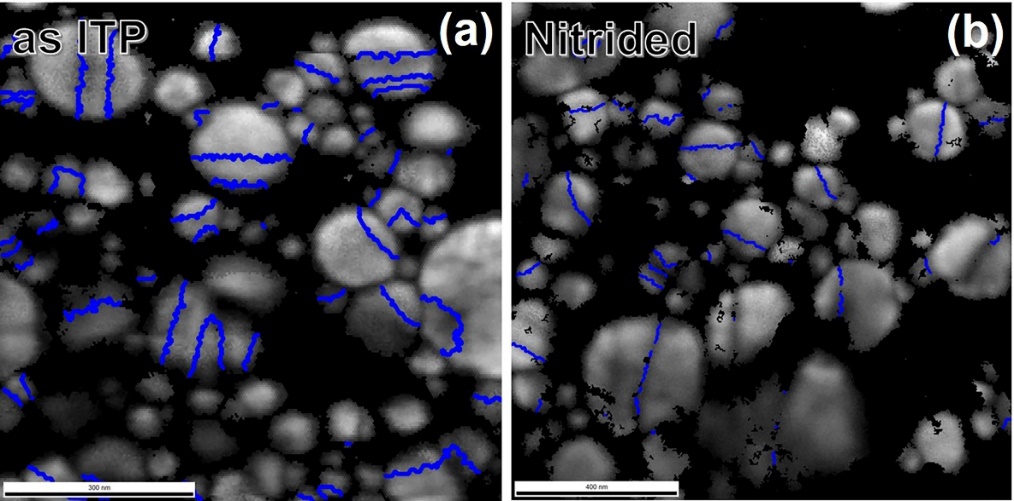


**Supplementary Figure S2 | Transmission Kikuchi diffraction (TKD) grain boundary (GB) maps of (a) as induction thermal plasma-processed and (b) nitrided FeNi NPs.** TSL-OIM 7 software was used to post-process the TKD raw data and the twin boundary was defined as the boundaries with angle between 65 to 75º. The twin boundaries are indicated by the blue lines. The fraction of NPs which contain twins is approximately 49.5% for the as induction thermal plasma processed FeNi NPs while it is 38.1% for the nitrided FeNi NPs. Please note that this value may be vary depend on the observation region in the GB map.


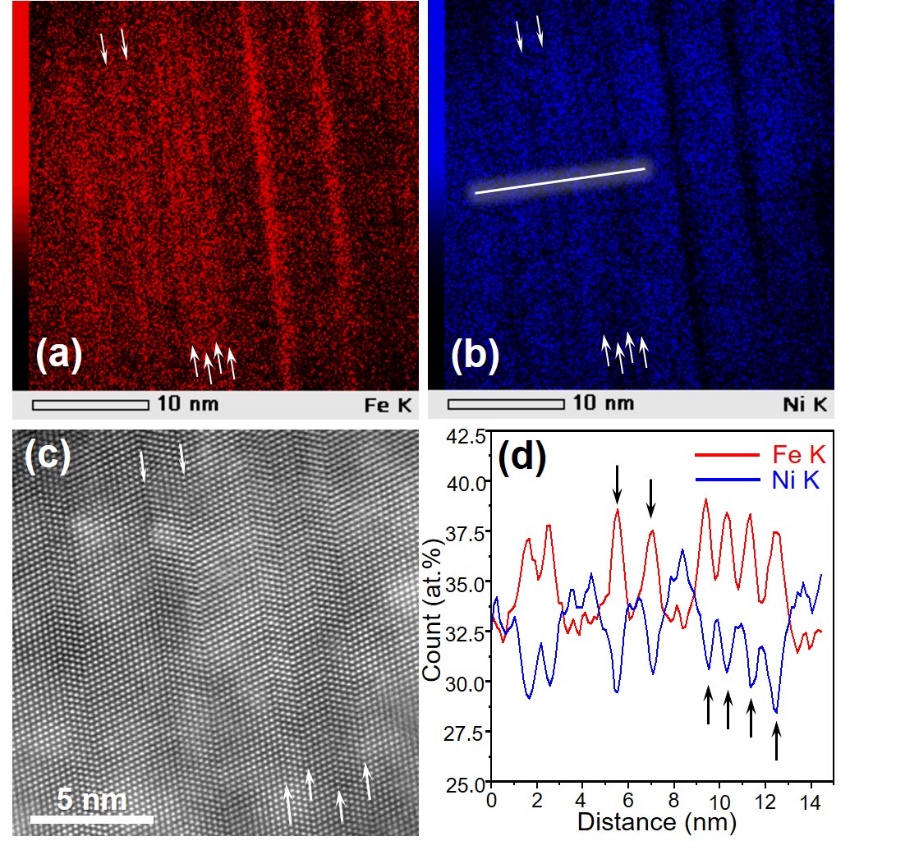


**Supplementary Figure S3 | Solutes segregations at the twin boundaries of nitrided FeNi NPs**. (a, b) High-resolution elements (Fe and Ni) distribution maps (EDS) of FeNi nitrided NPs. (c) Zoomed high-resolution TEM image of the nanotwins around the region indicated by the white line marked in (b). (d) A line elements scan profile across the nanotwins region as the white line marked in (b).


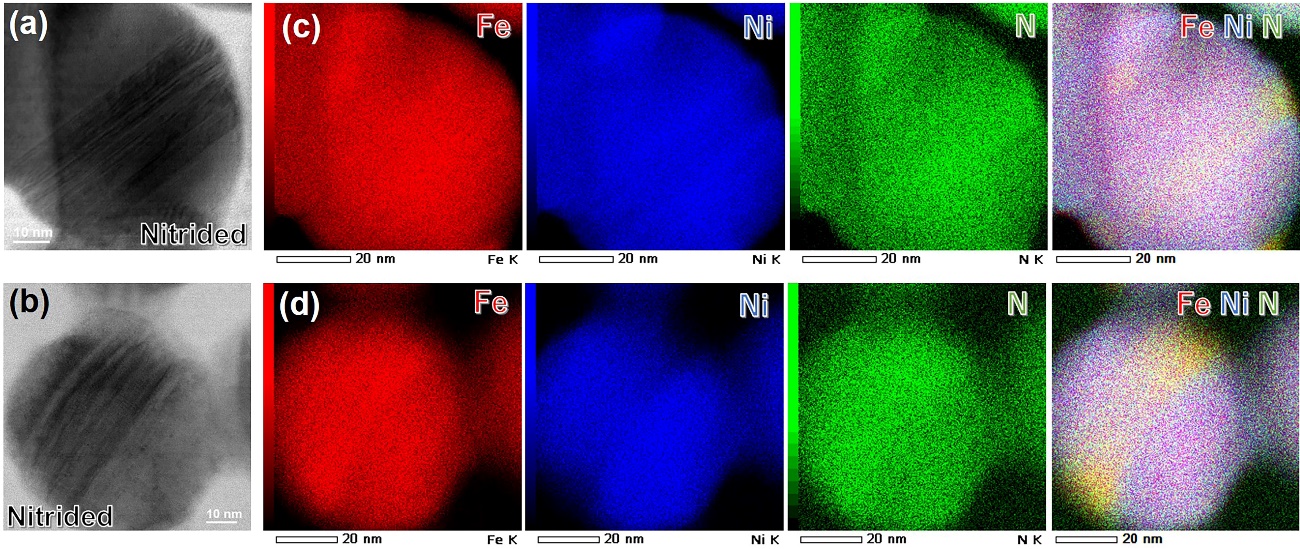


**Supplementary Figure S4 | Solutes segregation at the nanotwinned region of the nitrided FeNi NPs.** Low magnification ABF-STEM images of the nitrided FeNi NPs and the corresponding elements distribution maps (EDS). The stripe contrast in the ABF-STEM images originated from the diffraction of high density nanotwins. The corresponding line contrast in the EDS maps indicate that there is visible solutes segregation at the nanotwinned region. Interestingly, the N concentration is relatively higher at such nanotwinned regions.


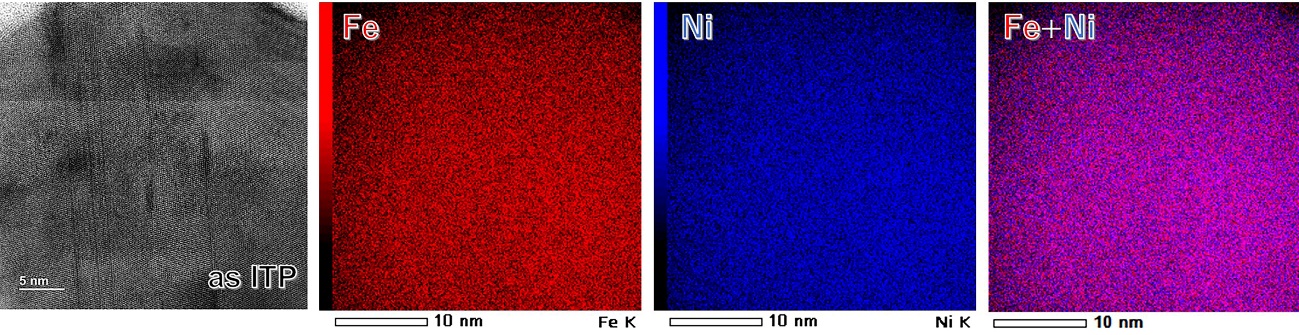


**Supplementary Figure S5 | ABF-STEM image and corresponding elements distribution maps of the induction thermal plasma processed FeNi NPs**. As high density of nanotwins (with typical width about 2 nm) can be detected in the ABF-STEM image, but there is no obvious solutes segregation along the nanotwinned regions.


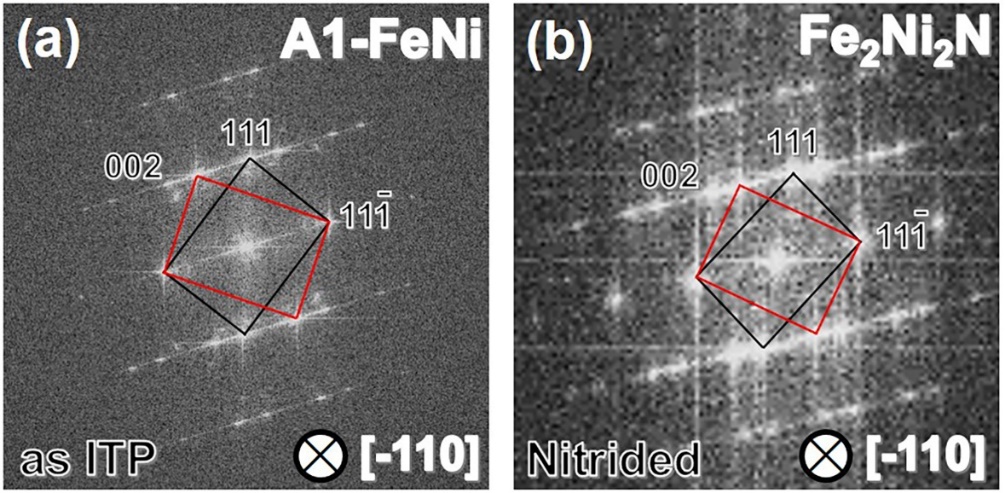


**Supplementary Figure S6 | Fast Fourier transform (FFT) patterns of (a) as induction thermal plasma processed and (b) nitrided FeNi NPs taken from Fig. 4 in main text indicating a typical twinning structure**. The zone axis for the FFT images is both [-110]. The two sets of FFT spots were marked with black and red rectangles and only the black rectangle spots were indexed. Accompany with the lattice spacing calculated from the high-resolution ABF-STEM images in **Fig. 4** in main text, the main phase is identified as A1-FeNi for the as induction thermal plasma processed FeNi NPs while it is Fe_2_Ni_2_N for the nitrided FeNi NPs.


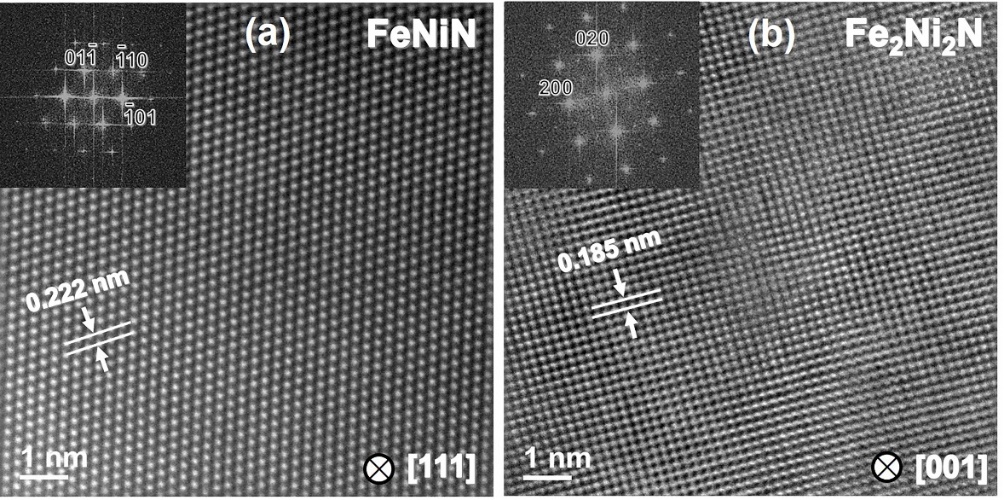


**Supplementary Figure S7 | High-resolution ADF-STEM image of nitrided FeNi NPs with (a) FeNiN and (b)Fe_2_Ni_2_N phases**. The zone axis is [111] for (a) while it is [001] for (b). Insets show the fast Fourier transformation (FFT) diffraction of the corresponding STEM micrograph. The exact phases identification was carried out with the lattice spacing in the ADF-STEM images and corresponding FFT patterns. The result of a mixture phases (FeNiN product phase and Fe_2_Ni_2_N parent phase) is consistent with the XRD results in Fig. 2c in main text.
